# Similarity between Red Blood-Corpuscles of Man and Those of Certain Other Mammals, Etc.

**Published:** 1875-05

**Authors:** J. J. Woodward

**Affiliations:** U. S. Army


### Selections.

### ON THE SIMILARITY BETWEEN THE RED BLOOD-CORPUSCLES OF MAN AND THOSE OF CERTAIN OTHER MAMMALS, ESPECIALLY THE DOG;

### CONSIDERED IN CONNECTION WITH THE DIAGNOSIS OF BLOOD-STAINS IN CRIMINAL CASES.

By J. J. WOODWARD, M.D., U. S. Army.

In his recent paper "*On the Value of High Powers in the Diagnosis of Blood-stains*,"* Dr. Joseph G. Richardson, of Philadelphia, affirms the possibility of distinguishing the blood of man from that of the pig, ox, red-deer, cat, horse, sheep, and goat, by the measurement of the red blood-corpuscles, even in dried stains, such as the microscopist is called upon to examine in criminal cases.

The circumstance that Dr. Richardson does not mention any animal whose blood-corpuscles cannot be thus distinguished from those of man, and the warmth with which he combats the prudent counsel which Virchow,†

* American Journal of the Medical Sciences, July, 1874, p. 102 ; also the Monthly Microscopical Journal, September, 1874, p. 130. This paper has attracted considerable attention. See, for example, the Lancet, August, 1874, p. 210 ; the Medical Times and Gazette, August 8, 1874, p. 151 ; and the London Medical Record, September 9, 1874, p. 560. The last of these Journals is the only one to raise a warning voice: "Dr. Richardson's paper is interesting, but we are afraid the question often put, 'What is the source of the blood in a stain ?' must go unanswered. In questions where capital punishment hangs on scientific evidence, that evidence must be of no doubtful or questionable nature."

† Rud. Virchow—Ueber die forensiche Untersuchung von trockenen Blutflecken—Virchow's Archiv., Bd. xii, (1857), S. 334.

Casper,* and Taylor,† in common with other experts,‡ have offered to enthusiastic microscopists in connection with this subject, led me, on perusing his paper, to fear he would be understood as teaching, in a general way, that it can be determined by the microscope with certainty whether a given stain is composed of human blood or not ; and this fear has been justified by some of the notices of his essay which have since appeared in the medical journals.

Now this subject is one which, from time to time, becomes of great importance in criminal cases ; and justice, no less than scientific accuracy, demands that the microscopist, when employed as an expert, shall not pretend to a certainty which he does not possess. I suppose no experienced microscopist, who has thoroughly investigated this subject, will be misled by Dr. Richardson's paper, but there are many physicians who possess microscopes, and work with them more or less, to whom a partial statement of facts on such a subject as this is peculiarly dangerous, and the object of the present paper is to point out to this class of readers that Dr. Richardson's statement of the case, even if all he claims be granted as true, is, after all, not the whole truth ; that there are certain mammals, among them the dog, the

* J. L. Casper—Handbook of forensic Medicine—translation of New Sydenham Society, London, 1861–5, vol i, p. 138, *et seq.;* also p. 198, *et seq.* See also the new and enlarged German edition of the same by Dr. Carl Liman—Practisches Handbuch der Gerichtlichen Medicin—5 Aufl. Berlin, 1871, Bd. II, S. 173, *et seq.*

† A. S. Taylor—The Principles and Practice of Medical Jurisprudence—2d edit. London, 1873, vol. i, p. 548.

‡ Among others, I may mention E. Brucke—Ueber die gerichtsarztliche Untersuchung von Blutflecken—Wiener Med. Wochenschrift, Jahrgang, 1857, S. 425. Hermann Friedberg—Histologie des Blutes mit besonderer Rucksicht auf die forensische Diagnostik—Berlin, 1852. Andrew Fleming—Blood-Stains—*American Journal of the Medical Sciences*, vol. xxxvii, N. S. (1859), p. 84. Wharton and Stille—Medical Jurisprudence—3d ed., Philadelphia, 1873, vol. ii, p. 696. M. Z. Roussin—Examen Medico-legal des Taches de Sang—*Annales d'Hygiene*, tome xxiii, (1865), p. 139. For an elaborate history of the growth of our knowledge on the subject up to 1860, the reader may consult B. Ritter—Zur Geschichte der Gerichtsarztlichen Ausmittelung der. Blutflecken, in Henke's *Zeitschrift fur die Staatsarzneikunde*, 1860. Drittes Vierteljahrheft, § 31. The chief authority in favor of the possibility of distinguishing the blood-corpuscles of man from those of other mammalia, is Carl Schmidt—Die Diagnostik Verdachtiger Flecke—Mitau u. Leipzig, 1848. I have not yet obtained a copy of this paper, but find abstracts of it in Schmidt's Jarbuch for 1849, p. 258, and Ritter's History, just cited. The reader will also find liberal extracts in Fleming's paper, cited above. The extravagant views of Schmidt are especially confuted by Brucke and Virchow in the papers cited above.

constant companion of man, whose red blood-corpuscles are so nearly identical in size with those of human blood, that they cannot be distinguished with any power of the microscope, even in fresh blood, much less in dried stains; and that, consequently, it is never in the power of the microscopist to affirm truthfully, on the strength of microscopical investigation, that a given stain is positively composed of human blood, and could not have been derived from the blood of any animal but man.

I must do Dr. Richardson the justice to state, at the outset, that these facts are well known to him, although, from motives of prudence, he has thought proper to be silent with regard to them. In a note dated October 19, 1874, in reply to one in which I informed him of my intention to write the present paper, he says: "I should be very much obliged to you if you would add to your remarks (in a foot-note or otherwise), that, in communicating with me, you found me fully aware of the difficulty of making anything more than a differential diagnosis, even in the cases I specified, and of the impossibility of distinguishing the blood of man from that of a monkey or dog, but that I had refrained from giving prominence to these facts," lest an improper use should be made of them in the defense of criminals.

I must, however, entirely dissent from this view of the matter. I cannot forget, that, on more than one occasion in the past, witnesses summoned as scientific experts have been so misguided as to go into courts of justice, and swear positively, on the strength of microscopical examination, that particular stains were human blood;* and I think the danger that others may do so in the future, to the prejudice of innocent men, is more to be feared than the possibility that an acquaintance with the true limits of our knowledge on this subject may sometimes be made use of in the unscrupulous defense of real criminals. I have, therefore, no hesitation whatever as to my duty, in speaking of this subject at all, to speak the whole truth, so far as it is known to me; and in so

* Passing by certain American cases, I may refer, in illustration of this statement, to the celebrated English case, Reg. *v.* Thomas Nation (Taunton Spring Assizes, 1857, p. 279), with regard to which the editor of the *London Medical Journal* has pithily said, that the testimony of the expert must be looked upon "as most disingenuous clap-trap, and rather what we might expect to hear at some popular lecture, where the 'wonders of the microscope' form the theme of declamation to a gaping audience, than the solemn asseveration on oath of a man of science in a court of justice."—*Med. Times and Gaz*, April, 1857, p, 366.

doing I am happy to say I follow the practice of many of the best writers on medical jurisprudence.

In the instance of the dog, it might at first sight be supposed, from the estimates of the average diameters of the red corpuscles in this animal and in man, as given by Gulliver and Welcker, the authorities most frequently cited in the modern text-books, that a certain small, but constant and measurable difference existed, which might serve as the basis of a distinction in legal cases. This inference, however, is not only contrary to the facts of the case, but an examination of the original essays of the authors cited shows that it is not borne out by their observations.

The mean diameter of the red corpuscles of man, according to Gulliver,* is 1-3200th of an inch (=.00794 millimetres), while that of the red corpuscles of the dog is 1-3542d of an inch (=.00716 mm.). With regard to his estimate for the human corpuscle, Mr. Gulliver† says: "We are only speaking now of the average size; for they vary like other organisms; so that in a single drop of the same blood you may find corpuscles either a third larger or a third smaller than the mean size, and even still greater extremes." According to this statement, the human red blood-corpuscles may vary in a single drop of blood from 1-4800th of an inch (=.00529 mm.) to 1-2400th (=.01058 mm.). Mr. Gulliver tells us further, in the same paragraph: "My own estimate of the average size has been deduced from numberless measurements, frequently repeated during the course of several years, of corpuscles quite fresh and swimming in the blood, and in various artificial mixtures, as well as in the dry state." I have not, however, been able to find, in those of his papers which I have examined, any of the numerical data from which this average size was deduced.

* George Gulliver, F.R.S.—Lectures on the Blood of Vertebrata—*Med. Times and Gaz.*, vol. ii, of 1862, p. 101, *et seq.*—On the Red Corpuscles of the Blood of Vertebrata, etc.; *Proceedings of the Zoological Society of London*, 1862, p. 91.—The Sydenham Society edition of the works of William Hewson, London, 1846, p. 216, *et seq.*—Appendix to Gerber's Elements of the General and Minute Anatomy of Man and the Mammalia, London, 1842, p. 31, *et seq.*—Observations on the Blood-Corpuscles or Red Disks of the Mammiferous Animals, *London and Edinburgh Philosophical Magazine*, vol. xvi, (1840), pp. 23, 105 and 195; also vol. xvii, pp. 139 and 325; also vol. xxi, (1842), p. 107. For a list of other papers referring to the blood-corpuscles of various animals, see the Works of William Hewson above cited, note to page 236.

† Med. Times and Gaz., vol. ii, of 1842, p. 157.

In the table of measurements appended to Gerber's Elements, in which for the first time he gave "mean or average sizes" (in previous papers he had only recorded "common sizes," occasionally supplementing these by the extremes observed), Mr. Gulliver explained his method of arriving at the average size as follows: "The common sized corpuscles are first set down, then those of small and large size, and lastly, the average deduced from a computation of the whole."* In this table the measurements for the common dog are given as follows:—†

| | | |
|---|---|---|
| Common sizes, | 1-4000 | of an inch. |
| | 1-3500 | " " |
| | 1-3200 | " " |
| Small size, | 1-4570 | " " |
| Large size, | 1-2900 | " " |
| Average, | 1-3542 | " " |

Where the "average" is simply the arithmetical mean of the several fractions given above, it can hardly, I think, be accepted as the true average size, since as much weight is given in this mode of calculating to the rarer as to the more frequent forms. Accordingly it is not surprising that we find in a former paper‡ measurements which do not accord very closely with this average: "Domestic dog, old mongrel, common diameter of corpuscles 1-4000th to 1-3200th of an inch." "Fox hound puppy, one day old, a bitch, 1-3000th, and 1-2666th, the most common diameter of the corpuscles." "Fox hound puppy, twelve days old, a bitch: most common diameter of the corpuscles 1-3000th and 1-2885th of an inch. Extreme sizes 1-4000th and 1-2666th." "Mongrel puppy, four months old, a bitch, all the following diameters common, viz.: 1-3693d, 1-3554th, 1-3429th, and 1-3200th." The measurements for the second and third of these animals are about as much larger than those for the human species as the others are smaller.

It is interesting to know just how Mr. Gulliver's measurements were made. He tells us he used a glass eyepiece micrometer so adjusted that the divisions had a value of 1-4000th of an inch each. "If one space and a quarter of this micrometer were occupied by a single globule, this would of course measure 1-3200 of an inch;

* Appendix to Gerber's Elements, cited above, p. 1.

† Loc. cit., p. 38.

‡ London and Edinburgh Philsophical Magazine, vol. xvi. (1840), p. 28.

if three equally-sized particles lying in a line, and touching at their edges, covered three spaces and a half, the diameter of each of these would be 1-3429th, if four spaces 1-3000th of an inch."* The objectives used were an eighth by Ross, and a tenth by Powell.† It is not stated whether these objectives were provided with the screw-collar adjustment for thickness of cover, but they probably were; and if so, doubtless all the measurements were somewhat vitiated, like others of the same date, by failure to allow for the variation in magnifying power produced by turning the screw-collar. Moreover, it must be clear, that practically the fractions of a division of the eye-piece micrometer were only *estimated*, for the case in which a number of "equally-sized" corpuscles would be found "lying in a line," and just "touching at their edges," without overlapping, must have been rare. As to the accuracy of the value assigned to the eye-piece micrometer, Mr. Gulliver himself says: "In the absolute accuracy of any micrometer applied to objects so extremely minute, it is difficult to place implicit confidence;" and he only claims "relative exactness" for his results.‡

Turning now to the original essay of Welcker, we find that his observations give even less support than those of Gulliver to the notion that the blood of the dog can be distinguished from that of man by the microscope. Welcker's measurements, as ordinarily quoted in the text-books, are .00774 of a millimetre for man, and .0073 for the dog. I find, in his original paper,§ that the mean for the dog was derived from the measurement of but ten corpuscles in the blood of a single terrier; the variations in this case being, minimum, .0065 mm., maximum, .0082 mm. Now, if we turn to the table ‖ of his own measurements of human blood, we find, that, in the last measurement of the blood of Dr. Schweigger-Seidel, fifty corpuscles gave the following results: mean, .00724 mm., minimum, .0051, maximum, .0085, in which case the mean is a trifle less than that found for the dog.

I would commend this table of Welcker to the study of those who may be disposed to underrate the diversity of size which may be observed among the human red

* Loc. cit., p. 24. † Loc. cit., p. 24 and p. 105. ‡ Loc. cit., p. 24.

§ H. Welcker—Grosse, Zahl, Volum, Oberflache und Farbe der Blutkorperchen, bei Menschen und bei Thieren. Zeitschrift fur Rationelle Medicin, 3te R., Bd. xx, (1863), S. 257.

‖ Loc. cit., p. 263.

corpuscles; the minimum measurement recorded in it is .0045 mm., the maximum .0097 mm.; the author remarks: "I have always, both in animals and in man, found the transverse diameter of the blood-corpuscles of one and the same individual vary from one-fourth to one-half of the mean measurement; and it appears that all the sizes lying between the two extremes are present in tolerably equal numbers, with the exception of the smallest corpuscles, which occur for the most part singly and at intervals."

I may mention, further, that the mean dimensions of the human red corpuscles so often quoted from Welcker, viz., .00774 mm., with a minimum of .0064 mm., and a maximum of .0086, were not derived from the whole of this table, but from four sets of measurements of his own blood only, of which two were dry preparations and two from the moist blood. He tells us that he selected the mean .00774 mm. because it *was* derived from his own blood, which he had used in a previous research on the number of the blood-corpuscles, and thought best, therefore, to use also in the computation of their volume, which is one of the chief subjects discussed in his paper. The mean of eight other measurements from five different individuals was .00768 mm. The blood of a chlorotic woman gave .00656 mm., as the mean of the corpuscles examined moist, and .00693 mm., as their mean when examined dry.

Welcker made his measurements with Kellner's System III, Ocular II, magnifying about 620 diameters, and by a delicately ruled eye-piece micrometer, each division of which, with the power used, had a value of .001723 mm., as determined by the stage micrometer: "A human blood-corpuscle fell within four or five of these divisions, while, on account of the great delicacy of the ruling, fifths or even tenths of a division could be estimated with tolerable exactness." The stage micrometer was a millimetre in one hundred parts, ruled by Lerebours, and which Welcker had verified by comparison with a standard scale in a manner which he describes in full, and which is worthy of study. He measured, as a rule, fifty blood-corpuscles from each sample, and these were not selected, but taken indiscriminately one after the other as they came under the scale while the specimen was being moved along.

Other observers besides Gulliver and Welcker have recorded minute differences in the average size of the red corpuscles of man and the dog. Thus Carl Schmidt* estimates the average diameter for man at .0077 mm., for the dog at .0070 mm. A. Kolliker † fixes the mean for man at .0033 of a Paris line (= .00751 mm.)—that for the dog at .0031 of a Paris line (= .00709 mm). On the other hand, Friedberg ‡ makes the blood-corpuscles of the dog the largest, stating that he finds the human corpuscles measure from .0058 to .0070 mm.—those of the dog from .0054 to .0080.

For myself, after repeated measurements of the blood of the dog, and of human blood. I can only say that I find no constant difference between them, whether the fresh blood or thin layers dried on glass be selected for measurement. The mean of fifty corpuscles taken at hazard is seldom twice the same, and sometimes that of human blood, sometimes that of dog's blood, is a trifle the largest.

The following measurements, intended to illustrate these facts, were made with a glass eye-piece micrometer ruled in two-hundred-and-fiftieths of an inch, and with such a magnifying power that each division corresponded to 1-50,000th part of an inch (.0005079 + mm.). The objectives used were an immersion 1-16th of Powell and Lealand, and an immersion No. 13 of Hartnack, either of which permitted the above value to be given to the divisions of the eye-piece micrometer by properly adjusting the draw-tube. The stage micrometer used in effecting this adjustment is an excellent one in 1-100ths and 1-1000ths of an English inch, in which the several hundredths and thousandths, as nearly as I can measure, are equal to each other, and the ten divisions of the latter value to any one division of the former—a quality in which the stage micrometers in the market are generally deficient. I have compared this micrometer with a standard scale ruled on silver—a centimetre in millimetres and tenths—the property of the United States Coast Survey, kindly loaned for this purpose by Mr. J. E. Hilgard, who assures me that it is "very accurate." I made several comparisons, both by means of an eye-piece micrometer and by the contact method described by Welcker. These comparisons showed that the divisions of my stage micrometer were nearly two per cent. (exactly 1.945 per cent.) larger than they ought to be, and this correction

* Op. cit.

† A Manual of Human Microscopic Anatomy. London, 1860, pp. 519 and 525.

‡ Op. cit.

was accordingly applied in adjusting the value of the eye-piece micrometer. The value assigned to the divisions of the eye-piece micrometer for these measurements cannot therefore, I think, differ from their absolute value by a quantity large enough to modify the results appreciably.

As the divisions represent a value twelve and a-half times less than that of the divisions of Mr. Gulliver's eye-piece micrometer, and more than three times less than those of Welcker's eye-piece micrometer, I did not find it necessary to estimate fractions of a division, as they did, but read the nearest number of whole divisions corresponding to each corpuscle. Fifty corpuscles, or about that number, were measured in each sample of blood. An assistant noted the number of eye-piece divisions corresponding to each corpuscle, as the measurements were made, and the mean was obtained in each case by adding together all the values and dividing by the number of corpuscles measured. Of course, the number of eye-piece divisions found only required to be multiplied by two to convert it into decimals of an inch. I endeavored at first to make these measurements with a dry Powell and Lealand's 1-50th of an inch, with the draw-tube so adjusted that each division of the eye-piece micrometer should equal one-hundred-thousandth of an inch, but I found the outline of the corpuscles, with this power, was not sharp enough to permit me to measure them as exactly as I wished, and I therefore gave the preference to the immersion objectives above mentioned.

Of course, in arranging for these measurements the effect of the screw-collar adjustment of the objective on the magnifying power had to be taken into account. This was done in the following manner: Some thin glass covers, not varying more than a thousandth of an inch from .012 of an inch in thickness, were selected from a lot of so-called 1-200ths of an inch covers by means of a suitable lever of contact.* Some blood being placed under one of these covers, the best adjustment of the screw collar for definition was found by trial. The stage micrometer, which is an uncovered one, was then temporarily covered with another of the selected thin glasses, and, being duly focussed upon, the desired value

* The instrument used was made by Stackpole & Bro., of New York, after the pattern of the instrument designed by Mr. Ross, which is figured in Carpenter on the Microscope, 4th ed., London, 1868, p. 203.

was given to the divisions of the eye-piece micrometer by the adjustment of the draw-tube, after which the measurements were proceeded with, and the screw-collar was not turned again until they were completed.

The following tables present the several means deduced from these measurements, in decimals of an inch, to which, for convenience, I have added the equivalent values in decimals of a millimetre. The number of corpuscles from which each mean was deduced is also given. The measurements made with the Hartnack No. 13 immersion are marked (H.), the others were made with Powell and Lealand's immersion 1-16th.

*Measurements of Human Red Blood-Corpuscles, from Five Individuals.*

| | Number of corpuscles measured. | Mean Diameter. Decimals of an English inch. | Decimals of a millimetre. |
|---|---|---|---|
| 1. Dr. W., dry . . . | 50 | .000304 | .00772 |
| 2. " " moist . . | 49 | .000292 | .00742 |
| 3. " " " (H.) . . | 50 | .000300 | .00762 |
| 4. " " " (H.) . . | 50 | .000289 | .00734 |
| 5. " McC., dry . . | 50 | .000288 | .00731 |
| 6. " " " . . | 50 | .000294 | .00747 |
| 7. " " moist . . | 50 | .000301 | .00765 |
| 8. Mr. W., dry . . | 50 | .000298 | .00757 |
| 9. " " " (H.) . . | 52 | .000297 | .00754 |
| 10. " T., " . . . | 50 | .000290 | .00737 |
| 11. " " " (H.) . . | 50 | .000292 | .00742 |
| 12. " B., " . . . | 50 | .000296 | .00752 |
| 13. " " " (H.) . . | 50 | .000297 | .00754 |

In each of these measurements of human blood, the great majority of the corpuscles ranged from twelve to seventeen divisions of the eye-piece micrometer—that is, from .00024 to .00034 of an inch. Out of the whole number measured, six were as small as ten divisions, and one as large as eighteen divisions; large and small forms were not searched for, however. The size most frequently measured was fifteen divisions, or .00030 of an inch.

*Measurements of the Red Corpuscles of the Dog, from Five Individuals.*

| | Number of corpuscles measured. | Mean Diameter. Decimals of an English inch. | Decimals of a millimetre. |
|---|---|---|---|
| 1. Mongrel terrier, dry . . | 50 | .000292 | .00742 |
| 2. Same animal, " . . | 54 | .000299 | .00759 |
| 3. Another mongrel terrier, dry (H.) | 50 | .000290 | .00737 |
| 4. Same animal, moist (H.) . | 50 | .000288 | .00731 |
| 5. Scotch terrier, " (H.) . | 50 | .000291 | .00739 |
| 6. Same animal, " (H.) . | 50 | .000289 | .00734 |
| 7. " " " (H.) . | 49 | .000287 | .00729 |
| 8. Spitz dog, dry (H.) . | 52 | .000285 | .00724 |
| 9. Black and tan, moist (H.) . | 50 | .000290 | .00737 |

In each of these measurements of dog's blood, precisely as in the case of those of human blood, the great majority of the corpuscles measured from twelve to seventeen divisions of the eye-piece micrometer (.00024 to .00034 of an inch). Out of the whole number measured, four were as small as ten divisions, but none larger than seventeen were encountered. As with the human blood, however, large and small forms were not searched for, but all the perfectly formed corpuscles brought into view by the movement of the stage were measured as they passed under the micrometer, without selection, until the required number was recorded. The size most frequently measured was fifteen divisions, or .00030 of an inch, precisely as in the case of human blood.

It will be observed that three of the above means for human blood, Nos. 1, 3 and 7, are a trifle larger than any of those of dog's blood, and two of the latter, Nos. 7 and 8, are a trifle smaller than any of those for human blood. All the other means for the dog are within the range of the values found for human blood, and the majority of them are each identical, even to the last decimal place, with some one of those found for man.

I may, moreover, remind the reader in this place, that the variations between the mean diameter assigned to human blood by different observers are quite as great as the variations recorded by any of them between the blood of man and the dog, or even greater. Passing by the older measurements, some of which, as a matter of curiosity, I have given in the foot-note,* I may cite, besides the measurements of Gulliver, .00794 mm., Welcker, .00744 mm., and Kolliker .00751 mm., which have been already quoted in this paper, the following values: Robin,† .0073

* A list of the more important of these older measurements will be found in the Mensiones Micrometricæ of R. Wagner (*Partium elementarium organorum quæ sunt in homine atque animalibus mensiones micrometricæ*. Erlangen, 1834). Most of these are included in the more complete list given by Louis Mandl (*Memoire sur les parties Microscopiques du Sang*, Paris, 1838, p. 10), from which I take the following, reducing the values which both Mandl and Wagner give in vulgar fractions of a Paris line to decimals of a millimetre. Leeuwenhoek (1673), .00902; *ib.* (1720), .01327; Jurin (1717), .00789; Tabor (1724), .00723; Senac (1749), .00820; Muys (1751), .01128; Weiss (1760), .01085; Della Torre (1763), .00301; Blumenbach (1789), .00789; Villar (1804), .00564; Sprengel (1810), .00902; Kater (1819), .00677; Bauer and Home (1818), .01504; Young (1819), .00451; Rudolphi (1821), .00902; Prevost and Dumas (1821), .00705; Edwards (1826), .00814; Hodgkin (1827), .00902; Wollaston, (1827), .00525; Weber (1830), .00525; Muller (1834), .00525 to .00902; Schultz (1836), .00667 to .00836; Wagner (1838), .00645 to .00752—Mandl, himself, gives .00800.

† Charles Robin. Note sur quelques points de l'anatomie et de la physiologie des globules rouge du sang. Journal de la physiologie, tom. i, (1858), p. 283.

mm., Harting, .0074 mm., Valentin,* .0071 mm., and Austin Flint, Jr.,† .00726 mm. (1-3500th inch).

I have thus shown that we are not justified, either by the facts of the case, or by the authorities supposed to favor the possibility of doing so, in attempting to distinguish between the blood of man and that of the dog, by the measurement of their red corpuscles. Mr. Gulliver himself, indeed, appears to have come to a similar conclusion, not only with regard to the dog, but several other animals, for he tells us that the corpuscles of the quadrumana, "differ but little from that of man, being only just appreciably, or sometimes not at all, smaller, both in the monkeys of the old and new continents," and that "in the seals, otters, and dogs, the corpuscles are about as large as in man."‡

I myself have not made systematic measurements of the blood of any of these other animals, and am, therefore, unable to speak as authoritatively with regard to them as I can about the dog. From Mr. Gulliver's detailed measurements, appended to Gerber's Elements, however, I am led to believe that there are several other animals whose blood, even in the fresh state, could not be distinguished by the dimensions of the red corpuscles from that of man. Among the domestic animals I may especially mention the rabbit and the Guinea-pig as belonging to this category. To these, besides most of the monkeys of both the old and new world, the seals, and the otters, we may add the kangaroo, the capybara, the wombat, and the porpoise. In the case of all these animals we not merely find that the "average size" calculated in Mr. Gulliver's peculiar way approximates dangerously to the average assigned to man, but the classic 1-3200th of an inch figures among the "common sizes" recorded by Mr. Gulliver for each.

The foregoing remarks and measurements refer especially to the fresh blood of the animals mentioned, and to thin layers quickly dried on glass, as is generally practiced in making preparations of blood for permanent preservation. In such preparations the corpuscles have almost exactly the size they possess in the perfectly fresh blood. The great majority of Mr. Gulliver's measurements were made upon blood prepared by this method,

* I cite the estimates of Harting and Valentin from Welcker's paper cited above, p. 258.

† The Physiology of Man, vol. i, New York (1866), p. 111.

‡ Proc. of the Zoolog. Soc., 1862, p. 96.

and at the time he appears to have regarded the results as the equivalent of measurements made on perfectly fresh blood. "In some instances," he tells us, "there was certainly a slight enlargement in the dried corpuscles, as compared with those seen in their own serum immediately after they were taken from the animal. In the greater number of trials, however, the sizes of the wet and dry discs corresponded accurately."* Twenty years later he seems to have modified this opinion somewhat, for he states, that, when the corpuscles of man and other mammalia were dried on glass, however quickly, they were usually just appreciably larger than in the "liquor sanguinis."† Welcker also found that the mean dimension obtained by measuring the corpuscles dried in a thin layer was apt to be rather greater than that obtained from the measurement of moist blood, and explained it by stating that "the very smallest more spherical corpuscles spread out a little in drying." He regards the difference, however, as so trifling that he uses measurements of dried specimens indiscriminately with those of moist in obtaining his averages. I myself am not satisfied that there is any constant difference, and find, on comparing the mean diameter of fifty corpuscles dry with fifty moist from the same individual, that sometimes the one, sometimes the other, is a trifle the largest. The dried corpuscles are very apt to be deformed, and often many of them are quite oval. If the long diameters of a number of such corpuscles are measured, the mean will be of course too great. I do not find it so if the measurement is confined, as it should be, to those corpuscles which have dried symmetrically and are quite circular.

How is it now with regard to blood dried *en masse*, when sprinkled upon weapons, clothing, wood, etc. Dr. Richardson admits in this case that a *slight* contraction takes place, but evidently regards it as too trifling to interfere with the diagnosis. Carl Schmidt, on the other hand, found that the blood-corpuscles under such circumstances contracted to nearly one-half their original size, and gives .0040 mm. as the mean diameter of the corpuscles of human blood thus prepared, while he assigns .0077 mm. as the mean of human corpuscles dried in thin layers

* Lond. and Edin. Philosophical Mag., vol. xvi, (1840), p. 25.
† Med. Times and Gazette, Aug., 1862, p. 158.

on glass.* It is not necessary for the purposes of the present paper to go into a detailed discussion of this question, for no one will pretend that it can be any easier to make the diagnosis of such stains than it is in the case of moist blood or of thin films dried on glass; and, if it is impossible in the latter case to ascertain by the microscope that the sample submitted is human blood, it would be absurd to hope to do better in the former. I cannot, however, refrain from expressing my conviction that Carl Schmidt was quite as accurate in measuring his samples as Dr. Richardson in measuring his, and that the latter has underrated the variations in size which the dried corpuscles may present under various conditions.

I may also call attention in this connection to the effect of water on the diameter of the corpuscles. Mr. Gulliver has pointed out, that, if "water be mixed with blood, the discs immediately become much enlarged and spherical, quickly losing their coloring matter; and yet, if the whole of this be thus removed, after a while the outlines of the discs, very faint indeed, may frequently be recognized, diminished considerably in diameter and apparently quite flat."† In another place he relates, that "some human corpuscles having an average diameter of 1-3429th of an inch, measured only 1-4800th of an inch after the whole of their coloring matter had been separated in this manner."‡ Suppose, now, the case of blood mixed with water and afterwards dried, as, for example, in the case of an unsuccessful attempt to wash away the blood while fresh?

In conclusion, then, if the microscopist, summoned as a scientific expert to examine a suspected blood stain, should succeed in soaking out the corpuscles in such a way as to enable him to recognize them to be circular discs, and to measure them, and should he then find their diameter comes within the limits possible for human blood, his duty, in the present state of our knowledge, is clear. He must, of course, in his evidence, present the facts as actually observed, but it is not justifiable for him to stop here. He has no right to conclude his testimony without making it clearly understood, by both judge and jury, that blood from the dog and several other animals would give stains possessing the same properties, and that neither by the microscope, nor by any other

* I quote from Fleming, op. cit., p. 111.

† Lond. and Edin. Phil. Mag., vol. xvi, (1840), p. 106.

‡ Ib., vol. xxi, (1842), p. 108.

means yet known to science, can the expert determine that a given stain is composed of human blood, and could not have been derived from any other source. This course is imperatively demanded of him by common honesty, without which scientific experts may become more dangerous to society than the very criminals they are called upon to convict.—*Am. Jour. Med. Sci.*, Jan., 1875.

